# Correction: DebtRank: A Microscopic Foundation for Shock Propagation

**DOI:** 10.1371/journal.pone.0134888

**Published:** 2015-07-31

**Authors:** Marco Bardoscia, Stefano Battiston, Fabio Caccioli, Guido Caldarelli

There are errors in the presentation of Fig 1 and Fig 2. Please view the complete, correct Fig 1 and Fig 2 here.

**Fig 1:**
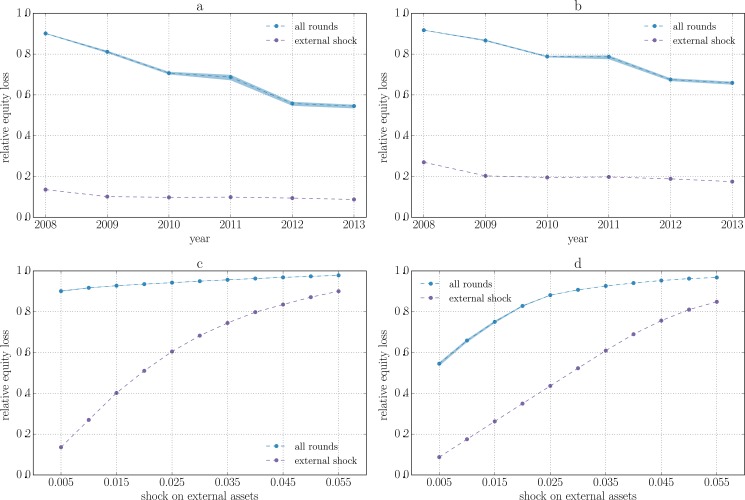
Relative equity loss for the system of 183 publicly traded EU banks between 2008 and 2013. All banks are subject to an initial shock consisting in the devaluation of their external assets by a factor α. The violet curves represent the relative equity loss that is directly due to the initial shock, while the blue curves include losses due to the contagion dynamics. Every point is the average over 100 reconstructed networks and the semi-transparent region covers the range between the minimum and maximum across the sample. α is fixed in the top panels and equals to 0.5% (panel a) or 1% (panel b). We see that the amplification effect is reduced from 2008 to 2013. Bottom panels refer to 2008 (panel c) and 2013 (panel d). We see that the relative equity loss saturates for large enough shocks. In 2008 the saturation already occurs for shocks as large as 0.5%.

**Fig 2:**
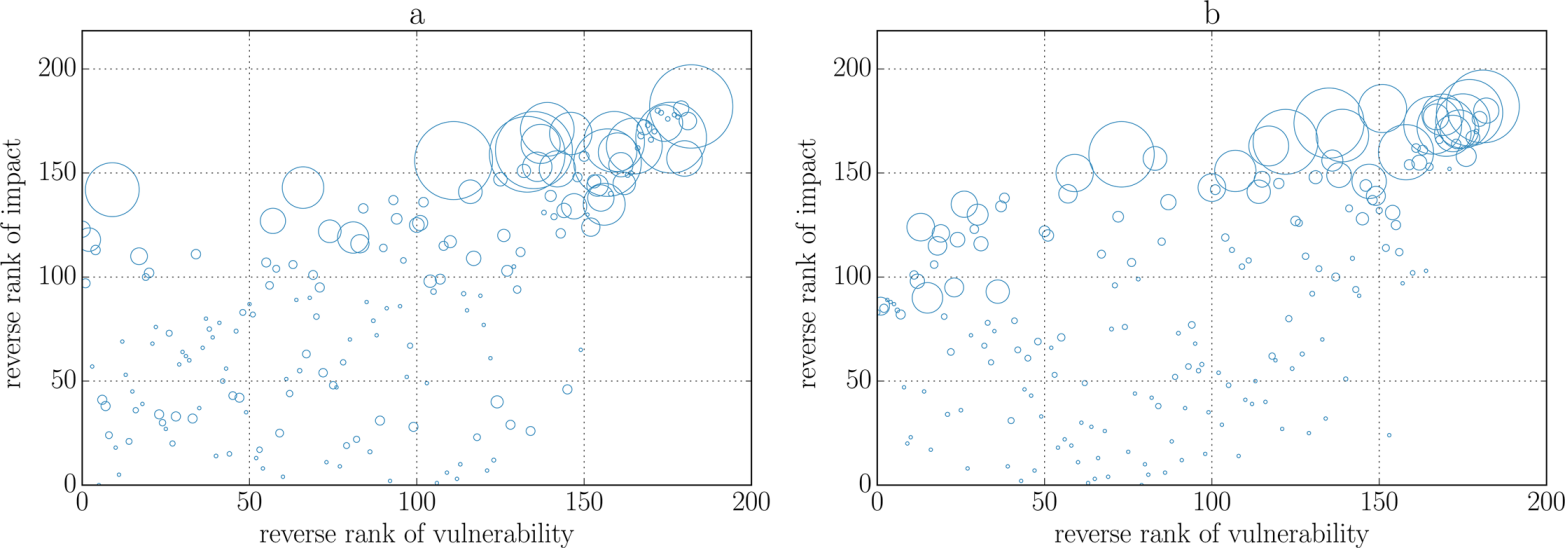
Scatter plot of impact and vulnerability (reverse) rankings in 2008 (panel a) and 2013 (panel b). An initial shock corresponding to a 0.5% devaluation of its assets is applied to one bank at a time, and the experiment is repeated for each bank. The impact of a bank is measured as the relative equity loss experienced by the system when that bank is shocked. The vulnerability of a bank is its relative equity loss averaged over all the experiments. In addition, we average impact and vulnerability across a sample of 100 reconstructed networks. Finally, we build reverse ranking (i.e. in descending order) of both quantities, so that larger values on both axes correspond to more impactful and more vulnerable banks. Bubble size is proportional to the total assets of the corresponding bank. The most dangerous banks are also the most vulnerable.

There is an error in the equation in the Data section. There is an incorrect subscript denotation in the second line. Please view the complete, correct equation here:

$$\begin{array}{l} \;A_{ij}^{\left(n\right)}=\frac{A_{ij}^{\left(n-1\right)}}{\sum_{j}A_{ij}^{\left(n-1\right)}}{\tilde{A}}_{i}\\ A_{ij}^{\left(n+1\right)}=\frac{A_{ij}^{\left(n\right)}}{\sum_{i}A_{ij}^{\left(n\right)}}{\tilde{L}}_{j}. \end{array}$$
